# The Central New York Homœopathic Medical Society

**Published:** 1887-12

**Authors:** Julius G. Schmitt

**Affiliations:** Secretary


### THE CENTRAL NEW YORK HOMŒOPATHIC MEDICAL SOCIETY.

DR. HAWLEY'S OFFICE, SYRACUSE, September 15th, 1887.

The meeting was called to order by President Dr. J. A. Biegler at ten A. M. The following members answered to the roll-call: Drs. J. A. Biegler, W. F. Clapp, Wm. A. Hawley, Stephen Seward, T. Dwight Stow, A. J. Brewster, J. R. Young, Harriet Dada Emmens, Leslie Martin, Julius G. Schmitt.

The report of last meeting was read and approved.

Dr. J. A. Biegler said he wanted to say a few words as to the disappointment he had felt in not doing more than he had as President of this Society, for he had intended to put his entire strength to the purpose of propagating pure Homœopathy, but sickness in his own family and the work the I. H. A. had put on his shoulders had prevented him from doing so, and he wanted these circumstances to be understood as the causes of his failure.

Secretary read Sections 16, 17, and 21 from the *Organon*, as having reference to the subject of the discussion for to-day.

The Secretary also read from a letter of Dr. R. B. Johnstone, of Germantown, the following passages:

"I should like to take part in the discussion, 'What Does Cure?' In my mind the thing is settled. It is that force which binds the molecules together and gives individuality to the substance used as medicine, and the higher the drug is potentized the further the molecules are separated, and the greater is the effect of that force which once bound them together. This same power may be seen every day. Take a lump of sugar, place it upon the dry tongue and no taste of sweet results, but as soon as water is added the molecular construction of the sugar breaks up, the force which bound them together is liberated (first potency), and we recognize that which we call sweet; thus a thousand illustrations might be mentioned.

"Remember me to all my fellow-members and say that the good cause goes on. I know of four young men, who have been practicing miserable mongrelism, who have been led from the error of their ways through my preaching and have joined the noble ranks of loyal Hahnemannians. They have discarded their tinctures and now carry only thirtieths and above. One of them said to me: 'Why, this is truly glorious! I cure my cases in one-half the time and leave nothing behind.' I cured lately a case of Psoas abscess with one dose of Hepar.$^{cm}$ and two doses of potentized 'glass,' which I am now proving is fully much more active than Silicea, and seems to cover many of the same symptoms. I am going to run it up to the 50m and the CM and will send you some."

*Dr. Hawley* said: The lately deceased Dr. T. L. Brown moved at our last meeting to discuss "What Does Cure?" and wanted to show that cures were almost entirely due to proper hygienic treatment and not so much due to remedies—a theory he favored so much. He maintained that homœopathic physicians did not pay attention enough to hygiene. I want here to relate a case of a man who was, and had always been, exemplary in his habits except his tendency for women, and in whom I failed to cure primary syphilis, which hardly ever happened to me before. The disease run into the secondary form and to paraplegia. I do not believe that syphilis ever can be cured by hygienic without medicinal treatment. Hahnemann describes what cures the best in Section 16. Air in a room cannot be driven out by fan, club, or any other crude instrument, but when you pour in that room gas from the gas-pipe the air in the room will be replaced by the gas, which is also an airy body. It is the similar alone that can fight the similar.

Dr. Biegler rejects Dr. Hawley's feeling humiliated in not curing his case of primary syphilis because he supposes every proper remedial means had been adopted; but, he continued, there are incurable cases, where nothing will have the desired effect. I want to relate a case, which shows plainly that medicines do cure. I saw, in council, a man sick with typhoid fever, with pronounced cerebral complication, whose sickness had withstood the best possible prescribing of the attending physician. There was a deep ulcer in right gluteal region, the color indicating Lachesis, besides a continuous desire to spit and persistent sleeplessness. After one dose of Lachesis, the patient became quiet and slept, after which consciousness returned. He needed no more medicine and fully recovered.

*Dr. Hawley:* I was called to see a portly, rather stout gentleman, fifty years of age, who complained of a terrific headache, especially in occiput; every bone of the body ached. Dark redness of face, jugular veins prominent. Calor mordax all over. No thirst. Dreams were worrying him, and his whole condition was aggravated by motion. He received *Bryonia* $^{40m}$ in four teaspoonfuls of water, one teaspoonful every half hour, followed by *placebo*. The next morning he was much better; continued *placebo*. He got along, improving for two days, when he took cold, and the redness of the face returned; Belladonna$^{40m}$ one dose dry was given. The next morning he complained that he had had a very bad night; there was a jumping pain in left side of head; he had not slept at all, and at ten A. M., when I saw him, was clamoring for relief. He received Spigelia $^{3000}$ in four teaspoonfuls of water, one to be taken every half hour until relieved. When I saw him at two P. M. the patient said that every dose he took aggravated the pains, so he did not take any more. I gave him Sac. lac., and saw him at four P. M., when I found him freer from pain, but the slightest movement seemed to start it again. At nine P. M. he told me that he had had an hour of very good sleep, but clamored for an anæsthetic, which was refused, and Sac. lac. given. Next morning much better, and he got well without any more medicine. This man had had sunstroke twice, in 1885 and 1886, and this summer, while exposed to the sun, fell in the street, but had sense enough to crawl into the shade. The aggravation by Spigelia showed that the medicine cured. I am fully convinced that, when we repeat too often, we have to fight our own remedies and not the disease. A single dose of Spigelia would, perhaps, have caused a slight aggravation and been followed by a speedy cure.

*Dr. Biegler:* I have been impressed by the same fact, and now never repeat unless the indications for it are without a shadow of doubt; and following this rule I have cured cases which before I could never have cured. A gentleman in South Carolina, whom I never had seen, complained by letter of severe headaches, which he had had for years. After, by frequent letters, he had been taught to describe his symptoms in a sensible way, I was able to prescribe for him intelligently, and he is now cured so that he can attend to his business, which imposes arduous duties on him. Yesterday I received a letter from him, in which he complains of bleeding piles that have been very bad since his headaches got better. Two and a half months ago I sent him a dose of Sulphur; finding that the symptoms he described corresponded with this remedy, I sent him Sac. lac. We can never tell how long a remedy will act.

*Dr. Young:* I was called to see a lady complaining of diarrhœa with asthma, calling for *Phosphorus*, which I gave in the two hundredth potency in water every hour. In the evening the report came that diarrhœa was better, but dyspnœa worse; sent her Sac. lac. Next morning she was all right. Was it the *Phosphorus* that aggravated the dyspnœa?

*Dr. Biegler:* I never repeat this remedy immediately, but, if I do, then generally after another remedy has been given.

*Dr. Clapp:* I treated, once, a case of typhoid fever where *Arsenic* was indicated; gave it in repeated doses without any favorable result. Another physician was called in council, who favored *Gelseminum*, but advised to stop the *Arsenic*, and to wait a while before I should give the other remedy. So I gave *Sac. lac.* In twenty-four hours patient improved, and there was no need for the Gelseminum. Arsenic, not being repeated, had a chance to act.

*Dr. Schmitt:* I want to tell you of a case which seems to prove that our remedies do really cure. I was called to see a young man who had been suffering off and on from attacks of renal colic, and who had been through various "scientific" courses of treatment. At the time I saw him he had taken the last dose of Epsom salt in the morning, and complained of severe pain in left lumbar region, left hypochondrium, and sometimes going down to bladder. He received a dose of Nux vomica$^{cm}$, and was soon relieved, improved steadily for six days, when, after returning from my office, he was taken with his old pain again. I was called at eleven P. M., and found him in perfect agony; he could not keep still, although lying on the painful side seemed to alleviate pain somewhat. There was frequent bilious vomiting. Rhus tox.$^{cm}$ was given, and in the morning there had been a slight relief, but by the time I saw him, at ten A. M., pains had grown worse again, going also to the right side. Berberis$^{cm}$, one dose. At that time he was talking of getting the help of two prominent allopathic physicians. I told him he could do, of course, as he pleased, but they would not tell him anything but what I did, and they would never cure him, whereas I would. In the evening the report came: "No better!" What should I do? I had promised the man a cure, and cured he must be. There were two other symptoms, to which I had not paid sufficient attention so far, viz.: *very strong smelling urine* and a *trembling in left lumbar region.*

Now I sent him Benzoic acid$^{cm}$, one dose.

When I saw him the next morning he had hardly any pain, and told me the following history: An hour after taking the first powder (Benz. acid$^{cm}$) he felt relieved and went to sleep, from which he was awakened by a sudden queer sensation; something moved down from left hypochondrium into bladder; he was cramped up for a minute, and since then he feels like newly born. On further inquiry, three little stones, a little larger than a pin's head, were produced. What I want to emphasize especially is, that the pain stopped first and then the calculi passed, showing the power of Benzoic acid to relax spasm of the ureter. In the course of a week he had a slight recurrence of the pains, which subsided rapidly under another dose of Benzoic acid. Since then no more attacks so far.

*Dr. Seward* said: Repeating of the dose depends upon the individual judgment of the physician.

Meeting adjourned at twelve M. until one thirty P. M.

*Dr. Biegler* called the meeting to order at one thirty P. M.

*Dr. Leslie Martin* asked whether any one present had used Johnstone's H. S. potencies.

*Dr. Hawley:* I have used Rhus tox.$^{cm}$ H. S. Johnstone, with great benefit.

*Dr. Schmitt:* I consider them very strong potencies, and I am afraid of using them with highly sensitive patients, on account of the aggravations they produce. I have a patient where I always have had to repeat high potencies of Swan's to produce an effect; not long ago he complained of symptoms which called for Rhus tox. I gave him one dose of the H. S. in the CM potency, and it acted like a thunderbolt. In his case, however, without aggravation.

*Dr. Hawley:* Cured a case of cancer of the uterus, which was also examined by Dr. Stephen Seward, with one dose of Sulphur$^{2c}$. He also spoke of Dr. Butler's paper on Sulphuric acid, and thought the black vomiting mentioned under this remedy ought to make it a valuable medicine for cancer of the stomach. Shortly after reading Dr. Butler's paper he saw a very sick man, who was vomiting a milky fluid; he had taken no nourishment but milk. The third night he saw him at nine P. M. and stayed with him until twelve P. M. There was a terrible distress in stomach, violent eructations, and examination revealed a thickening of the pylorus; finally the patient vomited more than a quart of a coffee-ground substance. He gave him at the time Placebos, and told his friends the next morning that, as the man had also rapidly lost in weight, the patient was suffering from cancer of the stomach. Dr. Hawley gave him *Arsenic*$^{40m}$ in water three times. Two other physicians, one an allopath, also examined the patient, and concurred in the Doctor's diagnosis. After the *Arsenic* he had once a spell of the same kind, but none since. For constipation he received a dose of *Nux vomica*$^{50m}$, which relieved. A week ago pains returned, but soon subsided after a dose of *Arsenic*$^{40m}$.

*Question:* "Is carcinoma curable?"

*Dr. Hawley* answers: Yes; the only trouble in not curing it lies in our hurry, not giving time enough for the highly dynamized remedy to develop its full action.

*Dr. Biegler*, in reference to Dr. Hawley's opinion, related the following case: A lady about seventy years old had been suffering for the last twenty years from what was called gastric difficulty, with obstinate constipation, so that her prevalent habit was to go three weeks without any movement, which was fixed. When I saw her, I found a hard oval mass between epigastrium and umbilicus, which I took for indurated mass of mesenteric glands, might have been the pyloric opening of stomach. Upon administration of indicated remedies, bowels commenced to move at first every week or ten days down to a regular daily, natural movement. This change occurred within six months. After this time tumor flattened out, as it were, and in course of a year disappeared. During existence of tumor a marked pulsation in epigastrium apparent to eye of bystander. After a year she suffered from frequent attacks of severe wind colic, resembling globus hystericus; these attacks at irregular times required several days' attendance. She also had frequent attacks of retching and vomiting—the latter clear mucous fluid; finally, perhaps after some imprudence in eating, she was one day taken with severe vomiting, resulting in throwing up of coffee-ground fluid, and finally, on my visit, she vomited a large washbowl full of thick, black fluid, attended with great prostration, amounting to collapse, such as one finds in severe cases of cholera. There was coldness of the surface, skin could be raised in folds, which remained standing, sunken eyes and features, marked thirst for cold water often. One dose of Arsenic$^{cm}$ was given, which resulted in immediate recovery, improvement setting in one hour after the dose, and the whole difficulty relieved in twenty-four hours. From that time to this, which is more than a year, she has remained a perfectly well woman.

*Dr. T. D. Stow* spoke of Hahnemann's theory of sickness and cure, as given in Section 16 of his *Organon*. While I lived in Geneva, he continued, intermittent fever abounded. One man having it for three years finally came to me to try Homœopathy. There were three prominent symptoms: Chill, seven to nine A. M., with desire for external warmth and coverings; intense thirst during chill, at no other stage. Vomiting, piercing headache, as if a spike were driven into the head. Paroxysms on alternate days. Ignatia$^{30}$ was given. The next chill was slight, and came at half past ten A. M., and the patient felt better altogether. He received one more dose of Ignatia$^{30}$ and never had an attack of ague again. There seemed no doubt that Ignatia cured.

A sailor, who had always suffered from malaria when coming into southern ports, came to me, complaining of chills commencing in the back, running up to head, nausea, bone pains, and other Eupatorium perfoliatum symptoms. This remedy cured him to such an extent that, when afterward exposing himself again to the above-mentioned noxious influences, he was not attacked with malaria any more. I have a case of osteosarcoma of the head of the tibia, where either resection of the head of tibia or amputation below the knee is indicated. He complains for a number of months of tasteless eructations, fullness of stomach after eating, tongue coated thin, white—worse in morning. He is anxious about himself and fearful. What is the remedy? I have opened the tumor at two points and inserted a silver drainage tube, which diminished its size. There is soreness of the tumor.

*Dr. Hawley:* Keep the knife away and you may have a chance to cure your patient. Select the remedy according to the symptoms. No remedy has been pushed so far in the provings as to cause osteo-sarcoma.

*Dr. Biegler:* You may find some symptoms, very valuable for a prescription, which have no reference to the tumor at all.

*Dr. Hawley:* There must be a constitutional taint in the man, otherwise he would not have developed such sickness.

*Dr. Biegler:* A young man was suffering from caries of the ulna. Part of the dead bone had been removed by an allopathic surgeon. At the time he came to me there was an abscess forming on the ulnar side of forearm near ulna; this I opened, and by probing found dead bone. I gave Silicea$^{6}$ (that time not using high potencies) every week or ten days, and cured him within three months. The patient, whom Dr. Schmitt also knows, is now a healthy, robust, middle-aged man.

*Dr. Schmitt:* While visiting a sick child in a family, I saw a boy of four years lying on a couch, groaning and moaning. Asking the father what the matter was with the boy, he told me that he had a swelling of the knee with abscesses, and that Dr. ———, an eminent allopathic surgeon, was going to reset the knee joint next week. I asked permission to look at the little sufferer, and found scrofulous inflammation of the right lower femur, four or five sinus openings discharging a stinking pus, the lower limb flexed as far as possible under upper limb. The boy in a continuous agony with pains night and day. Asked to prescribe for him. I gave him four doses of Silicea$^{mm}$, to be given once in two weeks. I never heard of the boy again until I saw him next summer playing with other boys. I examined his knee and found the leg semi-flexed, but so that he could step somewhat on his toes, the knee very little enlarged, and the openings all healed up. The proposed operation had not taken place.

*Dr. Biegler:* I want to relate the following case of intermittent fever: Chill, seven to nine A. M.; thirst during and before the chill; terrible bone pains, tertiary type. I gave a dose of Eupatorium perfoliatum$^{mm}$. The patient had no more chills, but came after ten days to my office complaining of constipation, etc., all symptoms pointing to Nux vomica. She received one dose of it in the CM potency; the next day she had the chills as bad as ever; then again a dose of Eupat. perf.$^{mm}$ cured. The Nux vomica defeated the action of Eupat. perf.

*Dr. Seward:* A lady suffering from swollen knee and great soreness in it to touch was greatly relieved by Calc. carb. and Calc. phosph.

*Dr. Hawley:* Is Nux vomica an antidote to Eupatorium perfoliatum? So far we know of no concordance to the latter remedy, and yet knowing the concording remedies to every one of our remedies, as given by Bœnninghausen in his *Pocket-Book,* is of the greatest use to us as prescribers.

*Dr. Schmitt:* I am glad that Dr. Hawley mentions this great work of Bœnninghausen, which ought to be studied more.

*Dr. Hawley:* I know of no better work in our homœopathic literature, and the International Hahnemannian Association ought to republish it.

*Dr. Biegler:* Much anxiety would be saved if in every case the first prescription were made with the utmost care. This first work, if ever so arduous, will do away with subsequent work, or will make it comparatively easy.

*Dr. Martin:* By following the example of Dr. R. C. Markham, of Jackson, Michigan, in using Bœnninghausen's *Pocket-Book* (see *Medical Advance* of July) I have been able to select my remedies with much greater certainty than before.

*Dr. Schmitt:* I have had the same experience, and have found that not alone the right remedy is found with more certainty, but that I have also enriched my knowledge of materia medica, as far as comparisons of different remedies are concerned.

*Dr. Stowe* moved that a committee of three be appointed to draft suitable resolutions on the death of Dr. T. L. Brown, of Binghamton, and they be published in the Binghamton daily papers, THE HOMŒOPATHIC PHYSICIAN, and the *Medical Advance* as soon as practicable.

Carried.

*The President* appointed as such committee Drs. Nash, Hawley, and Seward.

The Society then proceeded to the election of officers for the coming year, which resulted as follows:

President, Dr. T. D. Stow, Mexico, N. Y.

Vice-President, Dr. A. J. Brewster, Syracuse, N. Y.

Secretary and Treasurer, Dr. Julius G. Schmitt, Rochester, N. Y.

Censors: Dr. W. A. Hawley, Syracuse; Dr. Jos. A. Biegler, Rochester, N. Y.; Dr. E. B. Nash, Cortland, N. Y.

*Dr. Hawley*, who had suffered at the beginning of the meeting from a severe toothache, gave the history and cure, the latter resulting during the meeting. A tooth on right side, *which had been filled with amalgam*, feels too long, pains shoot up into face, worse from swallowing (Staph.), from touch and lying on affected side. Dr. Biegler prescribed Chinium sulph.$^{2c}$ with the happy result of seeing our good, old, sturdy friend in the afternoon as bright as a dollar.

*Dr. Brewster* related the case of a lady suffering from intermittent fever where Quinine in the first trituration had been given in vain. Chill, nine to ten A. M. Anguish, cramping pains in body and limbs, groaning, followed by *profuse* perspiration on chest and neck; thirst, but dysphagia. Paroxysms lasting three to four hours. During apyrexia great exhaustion. She received one dose of *Cimex*$^{600}$; next day chills slighter; the following day slighter still, and cure within five days.

As subject for discussion at the next meeting, "Contagious Diseases Caused by Acute Miasmata, according to Section 73 of the *Organon*," was selected.

The Society then adjourned to meet at ten A. M. on the third Thursday of December in Rochester, N. Y.

JULIUS G. SCHMITT,
*Secretary.*

